# P-1128. Effectiveness of KAIZEN Program for Reducing Spinal Surgical Site Infections Through Issue Identification via Descriptive Epidemiological Analysis and Enhanced Infection Control Measures

**DOI:** 10.1093/ofid/ofaf695.1322

**Published:** 2026-01-11

**Authors:** Hideki Kawamura, Shoko Arimura, Shinobu Uezono, Nao Murata, Aya Inamori, Hiroyuki Tominaga, Noboru Taniguchi

**Affiliations:** Kagoshima University, Kagoshima, Kagoshima, Japan; Kagoshima University Hospital, Kagoshima, Kagoshima, Japan; Kagoshima University Hospital, Kagoshima, Kagoshima, Japan; Kagoshima University Hospital, Kagoshima, Kagoshima, Japan; Kagoshima University Hospital, Kagoshima, Kagoshima, Japan; Kagoshima University Hospital, Kagoshima, Kagoshima, Japan; Kagoshima University Hospital, Kagoshima, Kagoshima, Japan

## Abstract

**Background:**

Addressing surgical site infections (SSIs) is challenging due to the involvement of multiple departments. This study aimed to assess the effectiveness of enhancing countermeasures, including issue identification via descriptive epidemiological analysis, in reducing SSIs in spinal surgery.

**Methods:**

We previously performed the bundle approach, including MRSA active surveillance with decolonization and cefazolin-based antimicrobial prophylaxis stewardship. In response to an increase in SSI cases detected through surveillance in 2018, the Infection Control Department conducted a descriptive epidemiological analysis to identify issues. SSIs were determined based on the NHSN definition and statistically analyzed using a risk index incorporating ASA classification, operative time, and wound classification.

**Results:**

Of the 11 SSI cases in 2018, five (45.5%) were caused by MRSA, though all patients were non-carriers preoperatively. Three patients (27.2%) were classified as ASA category 3, and seven (63.6%) had an operative time exceeding the 75th percentile. Intraoperative hypothermia (< 36°C) was observed in six patients (54.6%). Peak blood glucose levels >150 mg/dL within 48 hours postoperatively were identified in seven patients (63.6%), three of whom were non-diabetic. Postoperative blood glucose was not measured in three patients (27.2%).

Measures implemented to strengthen infection control bundles included intraoperative warming, blood glucose management for both diabetic and non-diabetic patients, and the use of a 0.35% povidone-iodine diluted solution for intraoperative wound irrigation.

The incidence of SSIs significantly decreased between 2020 and 2021 compared to 2018–2019 (0.6% [2/344] vs. 5.4% [17/315], P < 0.001), with a particularly notable reduction in cases with risk indices of 1 and 2 (0.8% [2/244] vs. 6.6% [13/197], P < 0.001).

**Conclusion:**

In addition to intraoperative intervention, the implementation of strengthened measures focusing on addressing hypothermia and hyperglycemia contributed to a reduction in the incidence of SSIs in high-risk spinal surgery. Problem identification through descriptive epidemiological analysis and multisectoral collaboration effectively strengthens SSI countermeasures.

**Disclosures:**

All Authors: No reported disclosures

